# Editorial: Phenotypes of myasthenia gravis, volume II

**DOI:** 10.3389/fneur.2023.1335308

**Published:** 2023-11-24

**Authors:** Hai-Feng Li, Nils Erik Gilhus, Qun Xue, Feng Gao

**Affiliations:** ^1^Department of Neurology, Xuanwu Hospital, Capital Medical University, Beijing, China; ^2^Department of Neurology, Haukeland University Hospital, Bergen, Norway; ^3^Department of Clinical Medicine, University of Bergen, Bergen, Norway; ^4^Department of Neurology, The First Affiliated Hospital of Soochow University, Suzhou, China; ^5^Department of Neuroimmunology, Henan Institute of Medical and Pharmaceutical Sciences, Zhengzhou University, Zhengzhou, China

**Keywords:** myasthenia gravis, phenotype, roadmap, biomarker, assessment

Following the first special topic in the *Phenotypes of Myasthenia gravis* (MG), the second special topic has been published. This special topic, including 3 original research articles and 1 brief research report, contributes to further understanding and assessment of MG phenotypes. Although fewer publications were included on this topic, the research progressed into a deeper and more comprehensive understanding and application of phenotypic analysis. Here, we briefly discuss these studies and present our reflections on a preliminary roadmap for MG phenotype studies.

## Phenotypic studies in this topic

A single-center, retrospective, 1:5 matched case-control study explored the relationship between a common comorbidity, type 2 diabetes mellitus (T2DM), and the risk of MG. One hundred and eighteen hospitalized MG patients were recruited for different comparisons with four datasets of different control groups, including the general population and inpatients hospitalized for non-MG and non-diabetes diseases. A conditional logistic regression analysis was used to test the risk of MG associated with T2DM. The risk of MG was found to be significantly associated with T2DM after a hierarchical analysis considering the onset of T2DM before or after the onset of MG, and other autoimmune diseases (Liu et al.). The same group previously found that diabetes mellitus aggravated the aberrant humoral immunity in MG patients by promoting differentiation and activation of circulating follicular helper T cells (1). The findings about MG and comorbidities in epidemiological studies need to be confirmed by more research on immunological mechanisms.

In a retrospective cohort of generalized MG patients who were consecutively recruited during the last 10 years, myasthenic crises were reported in a non-selected cohort from Istanbul, Turkey. Some interesting findings were noted. Thymic hyperplasia was found associated with a decreased risk of crisis. Furthermore, normal thymus was found in patients both with and without crisis in a similar proportion, which accounted for about 40% in both groups. In contrast to the expectations of the authors, most crises occurred in the summer, whereas more frequent and severe infections which may trigger myasthenic crisis occur in autumn and wintertime. The authors paid attention to COVID-19 infections during the summer of the pandemic. However, no patients developed a crisis after COVID-19 infection (Ozyurt Kose et al.). The limitation of this brief report is the sample size, being a single-center study. The relationship between myasthenic crisis and summer and thymic hyperplasia waits for confirmation from other patient cohorts.

A real-world evaluation of a smartphone-based research platform in characterizing precise symptom development during MG exacerbations was conducted in US MG patients who were representative of a moderate to severe MG phenotype, with frequent exacerbations, high symptom burden, and multiple comorbidities. There was a significant difference in the median MG Activities of Daily Living (MG-ADL) scores during self-reported exacerbation and non-exacerbation periods. Concordance between self-reported MG-ADL scores and exacerbation status was demonstrated. The dynamic changes in day-to-day symptom characteristics and severity, the daily step-counts as a measure of physical activity, and the general clinical characteristics of the patients were identified as essential variables to predict and evaluate the onset of MG exacerbations. Using all collected information, unsupervised machine-learning methods identified unique clusters of exacerbation subtypes. This smartphone-based research platform seemed to provide a convenient and accurate tool for characterizing symptoms in relation to MG exacerbations, and represents a paradigm in the patient-centered artificial intelligence research in the phenotypes of MG, which should also have high significance for daily clinical practice (Steyaert et al.).

In a gut microbiota and metabolite study, fecal samples were collected from 11 newly diagnosed untreated MG patients and 11 age- and sex-matched healthy controls. The microbial community richness and diversity were significantly lower in the MG group compared to the control group. Microbiota composition analysis revealed significant differences between the MG and control groups at phylum, family, and genus levels. A substantial decrease in the abundance of the genus Faecalibacterium was found in the MG group. Fecal metabolome analysis identified three up-regulated metabolites involved in amino acid metabolism and one up-regulated metabolite involved in lipid metabolism. A positive association was found between Faecalibacterium abundance and creatinine levels (Ding et al.). This study adds evidence regarding gut microbiota in the development of MG. More studies on the relationship between microbiota and the immune status of MG are awaited.

## A reflection on a preliminary roadmap for MG phenotype research

In editing the two special topics in the phenotypes of MG, we thought over again how to conduct phenotypic studies of MG, and provide our reflections on a preliminary roadmap for MG phenotype research.

### Clinical data elements for phenotype research in MG

A. Demographic data.

B. Optimize sensible methods for the precise timing of symptoms, comorbidities, aggravating factors, therapeutic adverse effects and other relevant factors or events.

C. Optimize data elements for the MG course (symptoms, standardized severity assessments, composite scores) and the accurate and convenient data collection methodology and smart tools.

D. Optimize basic data elements and derived data elements (i.e., composite measure derived from several basic elements, such as minimal manifestation status) that reflect the immune status of MG (e.g., clinical stability and the persistence of minimal manifestation status in relation to treatment intensity) (2, 3) and the functional efficacy of the neuromuscluar junction (e.g., response to AChE inhibitors or electrophysiological assessment of neuromuscular junction conduction capacity).

E. Optimize data elements for comorbidities and MG aggravating factors.

F. Optimize data elements for treatment intensity, compliance, and adverse effects.

G. Optimize data elements for psychological factors, emotional factors, and stressful life events.

H. Optimize data elements for special subgroups such as those with pregnancy, breastfeeding, infectious diseases, and malignanies.

I. Optimize data elements for pediatric patients, including growth and development.

J. Optimize data elements for thymus pathology and thymectomy, and related health consequences.

### Immunological data elements for phenotype research in MG

A. Optimize data elements for autoantibodies and disease-inducing antibodies of MG, i.e., antibodies against various epitopes of AChR, MuSK, and Lrp4 (4, 5). The reported results vary with different detection methods. The relationship between these culprit antibodies and MG phenotypes should be examined, including the association with severity and courses of MG (6, 7).

B. Optimize data elements for newly explored autoantibodies, especially regarding antigenic epitope, detection methods, method of processing raw data to form reported values, and the relationship to established antibodies or results with traditional methods for antibody measurement.

C. Optimize data elements for essential biomarkers of immunophenotypes of MG, including molecular and cytologic patterns of innate and adaptive immunity.

D. Assess the time-locked relationship between clinical data and molecular and cytologic biomarkers in monitoring the disease course and immune status of MG.

E. Optimize data elements for genomics biomarkers associated with the susceptibility and immune status of MG.

F. Optimize data elements for proteomics and metabolomics biomarkers associated with immune status.

G. Optimize data elements for microbiota associated with the susceptibility and immune status of MG.

### MG management and socio-economic analysis

A. Use patient-centered outcome measures, and actively integrate patient-involved study designs with traditional physician-led study designs (8).

B. Acquire accurate data through an optimal design of baseline and outcome measures, and operational quality control (9).

C. Develop performance measures for treat-to-target management in MG and assessment of potential factors for the refractory status.

D. Examine various treatment strategies and individualized pathways for the treatment of MG, especially the timing and combination of various interventions and their sequence.

E. Develop and evaluate more accurate assessing and predicting methods for short-term and long-term therapeutic efficacy.

F. Optimize data elements for socio-economic analysis for MG interventions in MG cohorts with different socio-economic background. Implement decision analysis, and explore the decision nodes, also with the inclusion of nest cost-benefit analysis.

G. Provide medical records including but not limited to the minimal information at each follow-up visit, including symptoms and severity, assessment of exacerbation, post-intervention status, detailed scores of each item of the commonly-used self-reported and physician-rated scales, immunotherapies and relevant duration between each follow-up, levels of disease-inducing antibody at irregular and/or prespecified intervals (e.g., 6 months in stable patients or 1 and 3 months after fast-acting immunotherapies for severe exacerbations), and major comorbidities and relevant management. The above information helps to provide the necessary information when the patients visit different physicians and to alert physicians and patients to some of their changes.

H. Develop the performance and quality control measures for the clinical classification, scale evaluation, post-intervention state and other standardized evaluation methods.

## Conclusion

Optimal MG treatment must adapt standard guideline recommendations to actual decisions for each individual patient. Modern medicine needs to be personalized but should at the same time be evidence-based, preferentially based on well-controlled scientific studies. However, such studies include patients with a variety of different characteristics. A key element for successfully combining general treatment standards with individual success is a precise evaluation of MG phenotype. Treatment guidelines should be adapted to phenotypically well-defined MG subgroups. Each patient should then be carefully evaluated regarding clinical and non-clinical parameters, leading to a precise phenotype assessment. The series of articles that we have edited in Frontiers of Neurology have illustrated the importance of such precise MG phenotyping, the consequences of having a specific MG phenotype, and the need for more research into defining phenotypic MG subgroups that are relevant for therapeutic response and safety.

## Author contributions

HFL: Conceptualization, Writing – original draft, Writing – review & editing. NEG: Conceptualization, Writing – original draft, Writing – review & editing. QX: Conceptualization, Writing – review & editing. FG: Conceptualization, Writing – review & editing.
